# A head‐to‐head comparison of four grading systems for oral epithelial dysplasia

**DOI:** 10.1111/his.15400

**Published:** 2024-12-17

**Authors:** Paul Hankinson, Mollie Clark, Hannah Walsh, Syed Ali Khurram

**Affiliations:** ^1^ Unit of Oral and Maxillofacial Pathology, School of Clinical Dentistry, Faculty of Health University of Sheffield Sheffield UK

**Keywords:** dysplasia grading, head and neck pathology, oral epithelial dysplasia, oral potentially malignant disorder, oral squamous cell carcinoma

## Abstract

**Aims:**

Oral epithelial dysplasia (OED) carries a risk of malignant transformation to oral squamous cell carcinoma. Clinical risk stratification for these patients is challenging, and reliant upon histological grading. The World Health Organisation (WHO) grading system is the current gold standard, although the binary system, two‐ and six‐point prognostic models have also been proposed. This study assesses the interobserver agreement and malignant transformation outcomes for these four grading systems.

**Methods and results:**

Up to 5 years of outcome data were collected for this retrospective cohort of 137 patients. Archived slides were reviewed by three pathologists, and grades for the WHO, binary, two‐ and six‐point systems were assigned. Interobserver agreement was assessed with Light's kappa coefficient. Kaplan–Meier and Cox regression survival analyses were used to assess the correlation of each grading system with malignant transformation. The WHO, binary, two‐ and six‐point systems had kappa coefficients of 0.42, 0.31, 0.17 and 0.41, respectively. All grading systems stratified lesions by malignant transformation risk, except the two‐point model. Moderate OED (WHO) did not show an increased risk of malignant transformation, while severe OED had a hazard ratio (HR) of 13.7 (*P* = 0.02). The high‐risk category for the binary and six‐point systems had HRs of 4.67 (*P* = 0.03) and 5.28 (*P* = 0.03), respectively.

**Conclusions:**

The interobserver agreement of the WHO, binary and six‐point systems is comparable. The six‐point and binary systems provided the most useful risk stratification. This study highlights the potential value of the six‐point prognostic model for OED grading, which has comparable performance with the current gold standard.

## Graphical abstract

This study examines four systems for grading oral epithelial dysplasia. WHO, binary and six‐point grading had comparable interobserver agreement. Six‐point and binary grading had more useful risk stratification compared to WHO. Two‐point grading performed poorly. Six‐point grading is at least equivalent to WHO grading (gold standard), although simpler to use.

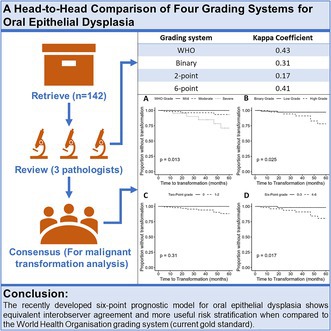

**Abbreviations**
CI: Confidence interval
IQR: Inter‐quartile range
LKC: Light's kappa coefficient
OED: Oral epithelial dysplasia
OSCC: Oral Squamous cell carcinoma
PVL: Proliferative verrucous leukoplakia
WHO: World Health Organisation

## Introduction

Oral epithelial dysplasia (OED) is a well‐established entity of the oral cavity which is associated with a risk of malignant transformation to oral squamous cell carcinoma (OSCC).^1^ The rate of malignant transformation varies greatly across the literature, with reports ranging from 1.4 to 50%.^1^, ^2^, ^3^, ^4^, ^5^, ^6^, ^7^, ^8^, ^9^, ^10^ Some clinical features of OED correlate with malignant transformation outcomes, as does treatment modality.^4^, ^6^, ^11^, ^12^ However, histopathological assessment of incisional biopsy specimens is central to patient management;^13^, ^14^ it is mandatory for confirmation of the diagnosis, and grading has been consistently linked to risk of malignant transformation.^3^, ^4^, ^5^, ^6^, ^8^, ^11^, ^15^

Currently, the gold standard for OED grading is the World Health Organisation (WHO) system,^1^ which categorises lesions as mild, moderate or severe. This grading system is essentially a professional qualitative assessment based on the degree of cytological and architectural atypia and its extent through the epithelium. This assessment is partially informed by dividing the epithelium into thirds, allowing for the allocation of a grade according to the vertical extent of cytological atypia.^1^ However, there is criticism of the value of thirds assessment of OED, which fails to appropriately account for clinical site variation, architectural atypia and other complexities of oral dysplasia grading.^15^, ^16^ As a result, the thirds assessment alone is considered inadequate with either significant cytological or architectural atypia, contributing to upgrading of a lesion.^1^ This complexity, in addition to the recognition of the large number (28) of cytological and architectural features described in the WHO classification, contribute to the difficulty of OED grading to the non‐specialist. It is not surprising, therefore, that this grading system is associated with variable interobserver agreement,^1^, ^17^, ^18^, ^19^ leading to attempts to simplify OED grading.

Alternative, feature‐specific grading systems have been proposed in order to address the complexity and poor interobserver agreement of the WHO grading system. One example is the binary grading system.^20^ This system differentiates between high‐ and low‐risk cases based on the number of architectural or cytological features present.^20^ It should be noted that this system is based on features of dysplasia described in the third WHO classification of head and neck tumours,^21^ which is now nearly two decades old. Strong evidence to support the binary system's superiority to WHO grading is lacking,^1^, ^10^, ^15^ despite some studies suggesting superior interobserver agreement.^9^, ^20^

**Figure 1:**
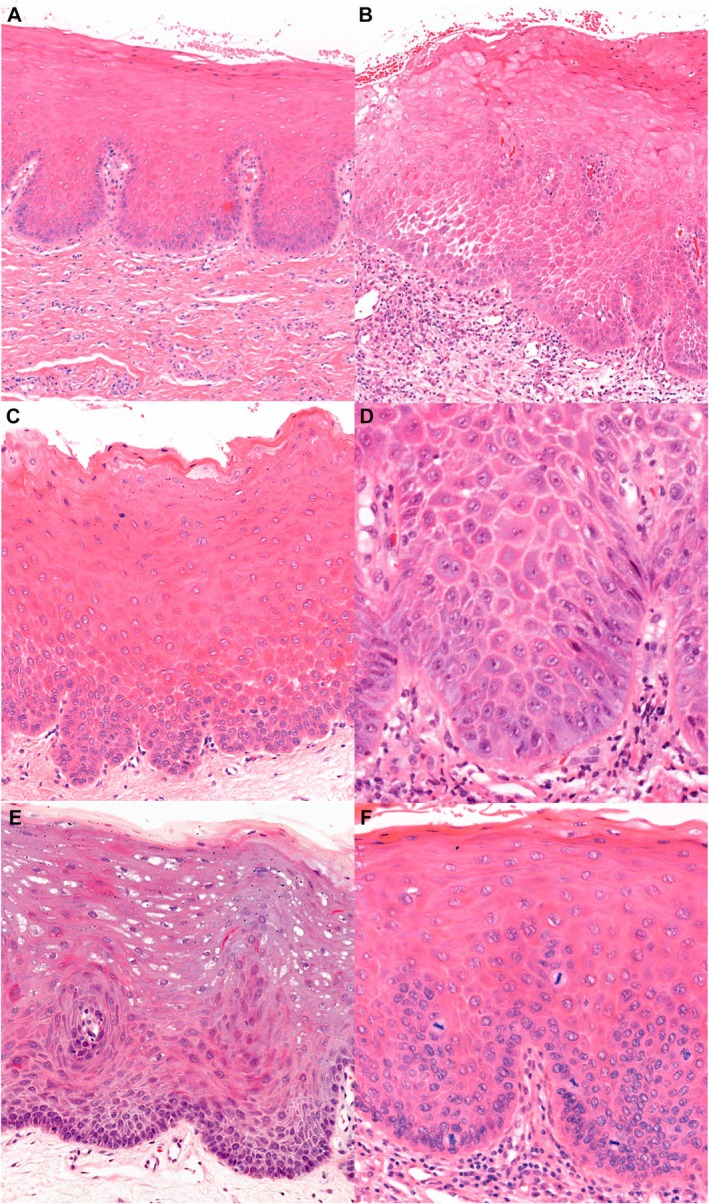
Example photomicrographs of the features in the two‐ and six‐point prognostic models. **A**, Bulbous rete processes. **B**, Loss of epithelial cohesion affecting the prickle cell layer. **C**, Loss of stratification affecting the basal cell layer. **D** Nuclear pleomorphism. **E**, Hyperchromatism of the basal cell layer. **F**, Supra‐basal mitotic figures in the prickle cell layer.

In recent years, other feature‐specific prognostic models for evaluating OED have been suggested.^22^ By correlating individual histological features of OED with malignant transformation and interobserver agreement, the ‘two‐point’ and ‘six‐point’ models were recently proposed.^22^ The six histological features of dysplasia found to be associated with an increased risk of malignant transformation, and included in the six‐point model were bulbous rete processes, loss of epithelial cell cohesion, loss of stratification, nuclear pleomorphism, hyperchromatism and supra‐basal mitotic figures (Figure 1). The first two features in this list had the highest interobserver agreement and were selected as the two features in the two‐point model. However, these new prognostic models require further evaluation to determine their clinical applicability.

This study aims to assess the interobserver agreement of the WHO and binary grading systems, as well as the two‐ and six‐point prognostic models. Moreover, it aims to assess each grading system's usefulness in stratifying patient malignant transformation risk.

## Materials and methods

This retrospective cohort study was completed with approval from the National Health Research Authority (Reference number: 18/WM/0335). A total of 150 patients with histologically proven OED were identified using histopathology records at a large tertiary care hospital. Archived histology slides were accessed for each patient, and the most representative tissue levels selected for examination. Patients were excluded if they had a diagnosis of OSCC before their earliest OED diagnosis, had Fanconi anaemia, did not have archived histology slides or sufficient tissue to determine if the histology represented OED or if, according to the team consensus, the histological diagnosis was not in keeping with OED. Patients with malignant transformation within 6 months of the initial diagnosis of dysplasia were not excluded. A total of 13 patients were excluded, but some of the included patients had multiple biopsies assessed. The total number of biopsy specimens included was 142. Follow‐up data were collected, including malignant transformation at the same site as the initial biopsy, time to malignant transformation, age, sex, location of lesion and any history of tobacco or alcohol consumption in excess of 14 units per week.

Each case was independently graded by three pathologists within a specialist oral and maxillofacial pathology department. Two consultant oral and maxillofacial pathologists (H.W., A.K.) and one senior registrar in oral and maxillofacial pathology (P.H.) were selected, all with significant experience reporting OED using the WHO and binary grading system. Each assessor allocated every case a WHO grade, binary grade and scores using the two‐ and six‐point prognostic models. Two‐point scores were also stratified into two risk categories, no points and one–two points, but only for the survival analysis. Six‐point scores were stratified into two risk categories, none–three and four–six points, for the interobserver agreement and survival analyses. Lesions were assigned a verrucous or non‐verrucous status based on previously described criteria.^16^ Unweighted Light's kappa coefficient (LKC) was used to calculate interobserver agreement.

To correlate grade with malignant transformation, a consensus grade was determined for each system. For WHO grading, full agreement was seen in 45% of the cases in the first instance and a consensus grade was agreed upon by the three assessing pathologists following discussion of the remaining cases. The three assessors were unanimous in their opinion for 47% of cases with binary grading, and 28% of cases with the two‐point score. For the remaining cases, if there was a majority grade this was selected; otherwise, a grade was agreed upon following discussion between the three pathologists. For six‐point scoring, 56% of cases had initial agreement for the allocation of the risk category, none–three and four–six points. For the remaining cases, a mean score was calculated using the six‐point scores in order to establish a consensus for the risk category. If a patient had multiple biopsies, the highest‐grade lesion was selected for survival analysis. Kaplan–Meier and Cox regression survival analyses were used to assess each grading system's correlation with malignant transformation. Cox regression included the covariates age, sex, tobacco history and alcohol consumption in excess of 14 units per week. All data analysis was performed in R (version 4.3.2) using the following packages: dplyr, ggplot2, irr, psy, boot, survival, survminer, ggsurvfit, and coxphf.

## Results

Following the exclusion of unsuitable participants, a total of 137 patients remained for assessment and grading of dysplasia (Table 1). Five patients had had two biopsies evaluated, raising the number of cases assessed to 142. Of the patients included in the analysis, 68 were male and 69 were female, with a median age of 62.3 years. In total, 84 patients (61.3%) had a history of tobacco use, while 52 (38.0%) consumed greater than 14 units of alcohol a week. The ventral tongue/floor of mouth was the most common site, followed by lateral/dorsal tongue, buccal mucosa, lip and gingivae in decreasing frequency. The majority of lesions were considered lower risk (mild/moderate for the WHO system, low risk for the binary system or none–three points for the six‐point model) among most of the grading systems, although only 19 patients (13.9%) scored no points on the two‐point model and 24 (18.2%) cases were considered verrucous. Median follow‐up was 37 months [interquartile range (IQR) = 14–60 months].

**Table 1:** Summary of demographic information, data on risk factors, OED site and grade allocated for each grading system

	*n* (%) or median (IQR), *n* = 137
Age (years)	62.3 (53.2–70.5)
Sex	
Male	68 (49.6)
Female	69 (50.4)
Smoker	
Yes	84 (61.3)
No	52 (38.0)
Alcohol consumption > 14 units per week	
Yes	52 (38.0)
No	83 (60.6)
Site	
BM	29 (21.2)
VT/FOM	46 (36.6)
Lateral/dorsal tongue	30 (21.9)
Gingivae	8 (5.8)
Lip	9 (6.6)
Palate	14 (10.3)
WHO grade	
Mild	46 (33.6)
Moderate	65 (47.5)
Severe	26 (19.0)
Binary grade	
Low risk	92 (67.2)
High risk	45 (32.8)
Two‐point grade	
0	19 (13.9)
1–2	118 (86.1)
Six‐point grade	
0–3	79 (57.7)
4–6	58 (42.3)
Verrucous	
Yes	25 (18.2)
No	112 (81.8)

BM, buccal mucosa; OED, oral epithelial dysplasia; IQR, interquartile ratio; VT/FOM, ventral tongue/floor of mouth.

A summary of the interobserver agreement for each grading system among the 142 biopsy specimens is shown in Table 2. The WHO system and six‐point model had the greatest interobserver agreement, with kappa coefficients of 0.43 [95% confidence interval (CI) = 0.34–0.52] and 0.41 (95% CI = 0.32–0.52), respectively. The binary system had lower agreement at 0.31 (95% CI = 0.21–0.40), although the difference was not statistically significant. The two‐point model had a significantly lower kappa coefficient than WHO or six‐point grading at 0.17 (95% CI = 0.09–0.24). Exclusion of verrucous lesions did not significantly impact the interobserver agreement for any of the grading systems.

**Table 2:** Interobserver agreement analysis of three independent graders. Light's kappa coefficients are given for each grading system with 95% confidence intervals

Grading system	All cases, kappa coefficient (95% CI)	Verrucous lesions excluded, kappa coefficient (95% CI)
WHO	0.43 (0.34–0.52)	0.45 (0.36–0.55)
Binary	0.31 (0.21–0.40)	0.37 (0.27–0.48)
Two‐point	0.17 (0.09–0.24)	0.18 (0.10–0.26)
Six‐point	0.41 (0.32–0.52)	0.41 (0.29–0.51)
Verrucous	0.51 (0.36–0.64)	

Left column, all cases (*n* = 142), right column only non‐verrucous cases (*n* = 117). CI, confidence interval.

**Figure 2:**
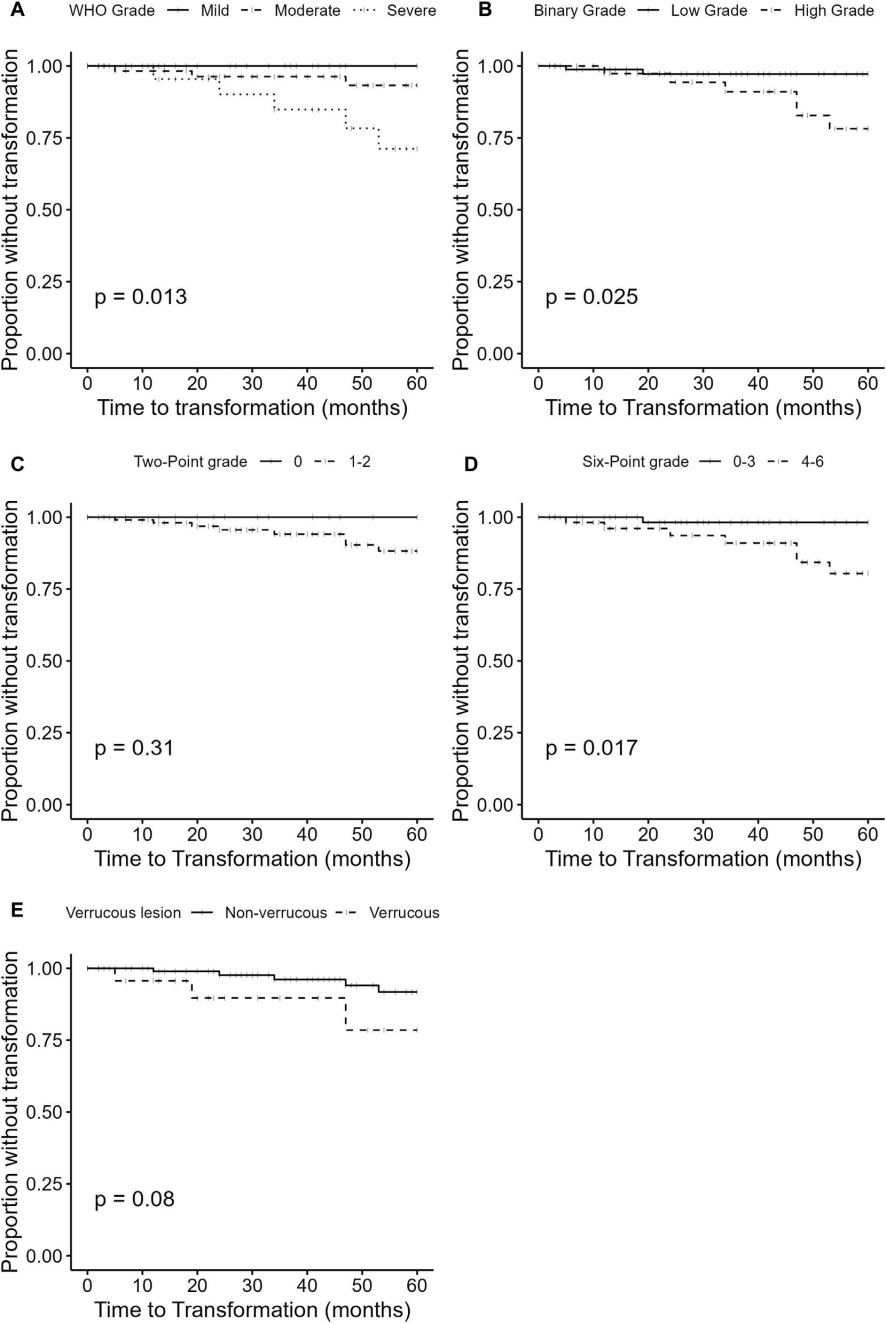
Kaplan–Meier survival curves of malignant transformation for each grading system and verrucous lesions. The log‐rank test was used to compare grade categories and generated *P*‐values. **A**, WHO system: solid = mild, dashed = moderate, dotted = severe. **B**, Binary system: solid = low risk, dashed = high risk. **C**, Two‐point model: solid = 0, dashed = 1–2. **D**, Six‐point model: solid = 0–3, dashed = 4–6. **E**, Verrucous lesions: solid = non‐verrucous, dashed = verrucous (*n* = 137).

Kaplan–Meier survival analysis (Figure 2) showed increased malignant transformation risk in the higher risk categories of the WHO (*P* = 0.013) and binary (*P* = 0.025) grading systems as well as the six‐point model (*P* = 0.017). Two‐point grading (*P* = 0.31) and presence of a verrucous architecture (*P* = 0.08) did not correlate with malignant transformation. Cox regression (Table 3), including the covariates age, sex, tobacco use history and alcohol consumption in excess of 14 units per week, confirmed these findings. Age, sex, tobacco use history and alcohol consumption in excess of 14 units per week did not contribute significantly to malignant transformation risk within this cohort. For the WHO grading system, the moderate category did not significantly increase transformation risk compared to the mild category, with a hazard ratio (HR) of 3.98 (*P* = 0.29). However, severe dysplasia had a HR of 13.7 (*P* = 0.02). The high‐risk category of the binary system had a HR of 4.67 (*P* = 0.03), while four or more points on the six‐point system had a HR of 5.28 (*P* = 0.03). Two patients had malignant transformation after the 5‐year follow up period, one at 65 months and the other at 106 months. Both these lesions were graded as moderate on WHO grading, low‐risk on binary grading, none–three points on the six‐point model and one–two points on the two‐point model.

**Table 3:** Cox regression analysis for each grading system, including the covariates of age, sex, tobacco history and alcohol consumption in excess of 14 units per week. Hazard ratios with 95% confidence intervals and *P*‐values are presented for each grade (*n* = 135)

Grading system	Grade	Hazard ratio (95% CI)	*P*‐value
WHO	Moderate	3.98 (0.37–541.95)	0.29
	Severe	13.71 (1.45–1833.75)	0.02
Binary	High risk	4.67 (1.14–26.14)	0.03
Two‐point	1+	1.78 (0.20–234.38)	0.67
Six‐point	4+	5.28 (1.14–50.20)	0.03
Verrucous	Verrucous	2.25 (0.47–10.10)	0.29

CI, confidence interval.

## Discussion

Effective grading systems for OED should be associated with a high degree of interobserver agreement, with little dependence upon pathologist experience. Of the grading systems examined in the present study, the WHO grading system and the recently proposed six‐point model showed the greatest interobserver agreement, with moderate agreement seen for both. Other studies examining inter‐rater agreement tended to find moderate agreement scores at best for WHO grading, with a wide variety of study designs employed. In 2020, Ranganathan *et al*. examined the interobserver agreement between six oral pathologists for a cohort of 72 cases of OED.^2^ They found that paired kappa coefficients ranged from 0.051 to 0.231, indicating poor to fair agreement.^2^ In a large cohort of 846 patients, Speight *et al*. demonstrated superior paired kappa coefficients for WHO grading between some observers of 0.706,^17^ although other paired observers had kappa coefficients as low as 0.251.^17^ Most studies sit within this range, with unweighted kappa scores of 0.22 to 0.644.^9^, ^20^, ^23^ In the present study, binary grading had a slightly lower (although not statistically significant) kappa coefficient than WHO or six‐point grading. A previous study showed comparable agreement for the binary grading system, with kappa coefficients of 0.049–0.326; however, this was still superior to WHO grading in their cohort.^2^ Most other studies also found binary grading to have higher interobserver agreement than WHO grading, ranging from 0.5 to 0.79,^9^, ^20^, ^23^ with most studies having a higher kappa coefficient for binary grading than this study. At present, to our knowledge, this is the first study to examine interobserver agreement for the two‐ and six‐point systems since their development.^22^ It should be noted that the kappa coefficients presented here represent agreement based on the risk categories (none–three or four–six points) for the six‐point model, and the absolute score for the two‐point model. Surprisingly, the two‐point model showed poorer performance when interobserver agreement was calculated on the risk categories of none and one–two points, rather than on the absolute score. These risk categories were selected as they are valuable prognostically, with the four–six‐points category having a significantly higher risk of malignant transformation when compared with none–three points in both this study and the study in which it was developed.^22^ Some studies have also found poor intraobserver agreement for the WHO grading,^1^, ^2^, ^19^ with binary grading achieving marginally better results.^18^, ^20^ However, no study has yet addressed the intraobserver variability of the two‐ and six‐point prognostic model. It is notable that, despite the three study pathologists working in the same team and reporting OED in a similar manner, there was still disagreement for all the grading systems. There may be many factors contributing to interobserver disagreement, and it is interesting that one study found the agreement on individual features was poorer than the agreement on overall grade.^18^ Perhaps differing understanding of what a feature is contributes to discordant grading.^18^ One further reason may be personal attitudes towards decision‐making and risk. Anecdotally, some pathologists will tend to assign a higher OED grade consistently. Finally, as already stated, pathologists often disagree with their own grading if a lesion is examined at a different time‐point, so it may not be surprising when they do not agree with each other.

The present study cohort showed relatively low overall rates of malignant transformation (6%) compared to those previously reported, although it is still within the wide range throughout the literature (3–50%).^6^ This may be a reflection of the higher proportion of low‐grade cases in this cohort. The majority (81%) of the cohort is made up of mild and moderate (WHO grade) dysplasia cases, with the minority allocated a severe grade (Table 1). Transformation rates of 0% of mild OED, 5% of moderate OED and 19% of severe OED were seen. In this study, WHO and binary grading, as well as the six‐point prognostic model, all stratified patients by risk of malignant transformation. Many studies have found that WHO grade correlates with malignant transformation,^5^, ^7^, ^9^, ^10^, ^12^, ^23^, ^24^, ^25^, ^26^, ^27^, ^28^ but most fail to demonstrate stratification between all three risk categories (mild, moderate, severe) or combine risk categories.^5^, ^7^, ^8^, ^10^, ^12^, ^23^, ^24^, ^25^, ^26^, ^28^ Moreover, a small number of studies even demonstrate higher rates of transformation in the lower‐grade categories.^7^, ^22^, ^29^ Concordantly, this present study found that moderate OED was not associated with a statistically significant increased risk of transformation compared to mild OED. However, it should be noted that the follow‐up for this study was 5 years. As OED may transform decades after initial presentation there may be cases, especially lower‐grade or verrucous lesions,^1^ that will transform but have not been identified in the follow‐up data for this cohort.

The findings of this study and others provide evidence that WHO grading cannot reliably separate OED into three distinct risk categories. Binary grading is generally successful in providing risk stratification of OED,^9^, ^10^, ^20^, ^23^, ^28^, ^30^ and is found to be comparable to WHO grading in several studies;^9^, ^10^ however, others failed to demonstrate risk stratification with binary grading.^26^ The greatest drawback of the binary system is the relatively high transformation rates within the low‐risk category in some studies^9^, ^23^, ^30^ although others, including this study, found a low rate of transformation in the low‐risk category.^26^, ^28^ Furthermore, it has not been tested on verrucous lesions or modified in light of the newly proposed features. Mahmood *et al*. found, while developing the six‐point prognostic model, that a score of four–six points had a higher risk of malignant transformation than none–one or two–three‐point categories, similar to the findings presented here.^22^ Unlike this previous study, however, the two‐point model failed to provide malignant transformation risk stratification.

All the grading systems discussed thus far have limitations in the interpretation of dysplastic lesions which show limited cytological atypia. For example, a verrucous pattern of surface keratin, or keratinisation which is abnormal for the site, may be regarded as OED in the correct context, even if all other typical features are lacking.^16^ Such lesions would not be deemed high risk by the criteria of any grading system, although it is well known that these lesions can form part of the proliferative verrucous leukoplakia (PVL) spectrum, which carries a high risk of malignant transformation of approximately 50%.^31^ While it is beyond the scope of the present study to provide detailed analysis of the grading of verrucous lesions, it is notable that good interobserver agreement was seen in the determination of verrucous versus non‐verrucous lesions. A significant association between the presence of a verrucous architecture and malignant transformation was not observed for this cohort. Further dedicated studies for the grading of verrucous lesions would be valuable.

Two cases in this cohort were found to have malignant transformation beyond the 5‐year period of this study, one as late as 106 months after the initial biopsy. Neither of these lesions were in the high‐risk categories of the WHO, binary or six‐point systems. This reinforces the need for long‐term follow‐up for patients, even with lower‐grade OED.

This study has identified comparable interobserver agreement between the recently developed six‐point prognostic model, the current gold standard (WHO grading) and the binary grading system. The promising performance of the new six‐point model within this study may be attributed to its objectivity and reduced complexity compared to the WHO and binary grading systems. The WHO system relies heavily upon the subjective experience of the reporting pathologist, an understanding of the numerous features of dysplasia and an understanding of how to interpret or disregard the vertical extent of changes throughout the epithelium as appropriate.^1^, ^16^ Moreover, the binary grading system relies upon identification of up to 19 histological features to stratify lesions into high‐ or low‐risk categories.^20^ The six‐point model is the simplest system to use, only requiring the counting of up to six features.^22^ Furthermore, categorisation of the six‐point model into two groups (none–three and four–six points) gave useful risk stratification, comparable to binary grading, despite having fewer features to assess. Two categories, rather than three, also avoided the issues faced by WHO grading in this study, with moderate OED failing to stratify malignant transformation risk from mild OED. The two‐point model, however, suffered from lower interobserver agreement than the other grading systems, and lacked the ability to stratify lesions by malignant transformation risk.

This study is limited by the cohort size and geographic distribution (single centre). Furthermore, the three reporting pathologists all originate from this single centre. The different methods employed for consensus grading may have introduced bias. Finally, as the two‐ and six‐point models were developed at this institution, some cases (*n* = 26) overlap between the study cohorts. Further studies, especially prospective in design and throughout broader geographic areas, would be beneficial for the comparison of these grading systems to determine which is the most reliable and useful in informing patient management decisions and prognosis.

## Conclusion

This study has demonstrated comparable interobserver agreement between the newly developed six‐point prognostic model of OED, the current gold standard grading (WHO) and binary grading. Moreover, six‐point grading provides useful risk stratification when classified into two risk categories (none–three and four–six points) and is equivalent to binary grading, with some advantages over WHO grading. While the six‐point prognostic model appears at least equivalent to the other, long‐established grading systems in this study it offers a simpler way to grade OED which does not rely upon specialist training or experience, as is the case for WHO grading. Future studies, ideally multicentre and prospective, should look to further validate this promising new prognostic model for OED.

## Conflicts of interest

The authors do not have any conflicts of interest to declare.

## Permission to reproduce material

No material has been reproduced from other sources.

## Data Availability

The data for this study is not publicly available.
